# Widespread dieback of riparian trees on a dammed ephemeral river and evidence of local mitigation by tributary flows

**DOI:** 10.7717/peerj.2622

**Published:** 2016-10-27

**Authors:** Caitlin M. S. Douglas, Mark Mulligan, Xavier A. Harrison, Joh R. Henschel, Nathalie Pettorelli, Guy Cowlishaw

**Affiliations:** 1 Environmental Dynamics Research Group, King’s College London, London, United Kingdom; 2 Institute of Zoology, Zoological Society of London, London, United Kingdom; 3 SAEON Arid Node, South African Environmental Observation Network, Kimberley, South Africa

**Keywords:** Drylands, Mortality, Dieback, *Prosopis*, *Faidherbia albida*, Intermittent rivers, Invasive species, Namibia, Ecohydrology, Human settlement

## Abstract

Ephemeral rivers act as linear oases in drylands providing key resources to people and wildlife. However, not much is known about these rivers’ sensitivities to human activities. We investigated the landscape-level determinants of riparian tree dieback along the Swakop River, a dammed ephemeral river in Namibia, focusing on the native ana tree (*Faidherbia albida*) and the invasive mesquite (*Prosopis* spp.). We surveyed over 1,900 individual trees distributed across 24 sites along a 250 km stretch of the river. General linear mixed models were used to test five hypotheses relating to three anthropogenic threats: river flow disruption from damming, human settlement and invasive species. We found widespread dieback in both tree populations: 51% mortality in ana tree, with surviving trees exhibiting 18% canopy death (median); and 26% mortality in mesquite, with surviving trees exhibiting 10% canopy death. Dieback in the ana tree was most severe where trees grew on drier stretches of the river, where tributary flow was absent and where mesquite grew more abundantly. Dieback in the mesquite, a more drought-tolerant taxon, did not show any such patterns. Our findings suggest that dieback in the ana tree is primarily driven by changes in river flow resulting from upstream dam creation and that tributary flows provide a local buffer against this loss of main channel flow. The hypothesis that the invasive mesquite may contribute to ana tree dieback was also supported. Our findings suggest that large dams along the main channels of ephemeral rivers have the ability to cause widespread mortality in downstream riparian trees. To mitigate such impacts, management might focus on the maintenance of natural tributary flows to buffer local tree populations from the disruption to main channel flow.

## Introduction

An improved understanding of the ecology of intermittent rivers, which comprise over half the length of the global river network, has recently been highlighted as a priority for scientists, policy makers and managers (Datry, Larned & Tockner, 2014). Ephemeral rivers are particularly dry intermittent rivers. They are dry for the majority of the year, and only flow for all or part of their length during and after rain (Tooth & Nanson, 2011). Large ephemeral rivers, primarily associated with drylands, possess alluvial aquifers which support abundant vegetation, including woodlands, which are distinct from the sparse vegetation of the surrounding desert environment. These woodlands in turn support human and wildlife populations, providing important resources such as food/fodder, shelter, construction materials and medicine. Ephemeral rivers have thus been described as ‘linear oases’ (Jacobson, Jacobson & Seely, 1995). However, the societal and ecological value provided by ephemeral rivers and their associated woodlands is often overlooked. As a result, these ecosystems are under threat from a variety of pressures (Steward et al., 2012). The purpose of this study is to explore the impact of three potential anthropogenic threats on woodland survival along an ephemeral river: the disruption of river flow from damming, human settlement and invasive species.

The primary threat we consider is the disruption of river flow from damming. Dams, along ephemeral rivers, are built to obtain and store water and their presence can alter water flow, reducing the magnitude and frequency of flow events (Hughes, 2005). River flow is an important element of ephemeral river ecosystems in that water and nutrients are brought to trees and groundwater is recharged, supporting the trees for the rest of the year when the river is not flowing (Jacobson, Jacobson & Seely, 1995; Schachtschneider & February, 2010; Jacobson & Jacobson, 2013). Infiltration of river flow is the primary source of groundwater recharge along ephemeral rivers (Morin et al., 2009). Flows also help to regulate soil and groundwater salinity (Jolly, Walker & Thorburn, 1993), preventing adverse salt accumulations (Waring & Schlesinger, 1985). Disruption to these processes has the potential to be highly damaging to tree populations. However, flows are not always beneficial. Flows of long duration can cause mortality due to oxygen depletion in the root zone, and large flows can cause trees to be washed away (Friedman & Auble, 1999; Friedman & Lee, 2002). Thus, flow disruption from damming may increase or decrease tree mortality depending upon the flow and channel characteristics, the tree species and its location in the channel. A further complication is that tree death from such effects may be more severe in areas of greater climatic stress, such as hotter, drier, areas where trees experience higher water demands (Niklas, 1992). Unfortunately, while the downstream effects of dams on riparian tree health have been well-documented along perennial rivers (Rood & Mahoney, 1990; Kingsford, Lemly & Thompson, 2006), their impact along ephemeral rivers has yet to be systematically assessed.

One potential buffer to these effects may be provided by tributaries. Tributaries provide an additional source of water, nutrients and sediments to the main channel of the river (Rice, Rhoads & Roy, 2008). These inputs have important implications for physical processes along perennial rivers, and the role of tributaries within a river system can become more important after the main channel has been dammed upstream (Petts, 1980; Benn & Erskine, 1994; Rice et al., 2008). There is increasing recognition that tributaries also play an important role in ecological processes, but research on this topic has so far focused on aquatic animals in perennial rivers (Rice et al., 2008). To our knowledge, very little work has been conducted on the role of tributary confluences in regards to tree population dynamics. In ephemeral rivers, tributary effects of any kind have yet to be documented.

The additional threats we consider in this study are human settlement and invasive species. In the first case, human settlement in drylands typically impacts tree populations through livestock browsing and water abstraction. Browsing is more likely to affect tree recruitment than dieback, but severe browsing could potentially lead to mortality in certain smaller trees (Bergström, 1992). In contrast, water abstraction can have profound impacts on tree survival by reducing groundwater levels below the rooting depth (Le Maitre, Scott & Colvin, 1999). In the second case, invasive alien trees can be associated with dieback in native trees (Iponga, Milton & Richardson, 2008; Schachtschneider, 2010; Shackleton, Le Maitre & Richardson, 2015). This can occur through a variety of mechanisms, including direct competition for water, nutrients and light, or through indirect means such as allelopathy or the alteration of soil characteristics (Davis, 2009).

Our study focuses on the Swakop River, Namibia, an ephemeral river that is subject to all three threats (Jacobson, Jacobson & Seely, 1995). We begin our analysis by assessing the scale of dieback for the native ana tree (*Faidherbia albida* (Delile) A. Chev.) and invasive mesquite (*Prosopis* spp. L.).

Mesquite has a reputation as an aggressive invader and is regarded as the terrestrial invasive taxon of greatest concern in Namibia (Bethune, Griffin & Joubert, 2003). In particular, mesquite is thought to outcompete native trees for water, leading to dieback (Robertson & Woodborne, 2002; Schachtschneider & February, 2013).

Dieback is assessed through canopy dieback, which varies between 0% (in completely healthy trees) to 100% (in dead trees). On describing high levels of mortality in both species, we go on to test five hypotheses about which threat processes (a disrupted river flow from damming, human settlement and/or invasive species) might explain this dieback. Like other large ephemeral rivers, only very sparse data are available for the Swakop River on the frequency and volume of flow, groundwater depth, groundwater salinity, and how these have varied over the lifetimes of the riparian trees of interest. Consequently, we focus on alternative landscape-level variables that should nevertheless reflect the influence of these parameters.

Our first three hypotheses test for a potential impact of disrupted river flow from damming. First, we investigate how tree survival relates to position in the channel, using a combination of horizontal distance and vertical elevation from deepest point in the channel, since these variables provide an indication of exposure to flow and access to groundwater. Specifically, as horizontal distance away from the channel increases, tree dieback may either increase, as trees further away from the channel are less likely to receive the benefits of flow (Hypothesis H1.1a), or decrease, due to protection from large flows and/or water-logging (H1.1b). Similarly, as elevation above the channel increases, tree dieback may either increase, due to trees receiving less benefit from the flow that tends to occupy the deeper channel region (H1.2a), or decrease, due to protection from large flows and water-logging (H1.2b). Second, we assess how tree survival relates to local climate; in hotter, drier, areas trees already experience higher water demands and therefore may be less able to cope with any reductions in channel flow and/or groundwater decline (H2). Third, we test the hypothesis that tributary flows buffer against disrupted flows in the main channel, by assessing whether observed tree dieback is lower below tributary confluences (H3). Our final hypotheses relate to human settlement and invasive species, respectively. Fourth, because human settlement may have adverse impacts on tree survival, we test the hypothesis that dieback is more extensive in the presence of human settlement (on private and communal land) than in its absence (protected park land) (H4). Fifth, because the invasive mesquite is believed to have detrimental effects on the native ana tree, we test the hypothesis that ana tree dieback is greater where mesquite is more abundant (H5).

## Materials and Methods

### Study area

The Swakop River is one of Namibia’s twelve major ephemeral rivers. For the majority of the year, Namibia’s ephemeral rivers consist of dry sandy riverbeds, with flows only occurring after precipitation. The duration of flows can vary considerably; typically lasting between a few days to several weeks, but in years with exceptional amounts of rainfall for a few months (Jacobson, Jacobson & Seely, 1995). Although Namibia’s major ephemeral rivers are without flow for much of the year, groundwater is contained throughout the year in an alluvial aquifer.

From its headwaters in the Khomas Hochland Plateau the Swakop River flows westward 460 km through the Central-Western Plains to the coast (Table 1). There is a near linear decline in precipitation along the river’s length: the headwaters receive 475 mm of precipitation annually but the coast receives nil, and 80% of the catchment experiences less than 100 mm per annum (Jacobson, Jacobson & Seely, 1995). It is therefore the precipitation in the river’s headwaters which drives much of the Swakop’s flow. This precipitation falls in the austral summer. Although the inter-annual variability in rainfall is high, the long-term annual rainfall has remained stable over the last 100 years (*e.g.* for Windhoek, in the southeast of the catchment, see Mendelsohn et al., 2009; for Otjimbingwe, in the centre of the catchment, see Ward et al., 2000). Groundwater salinity increases linearly downstream (Gevers & Westhuyzen, 1937; Bittner, Kuells & Marx, 2010), although there may be small fluctuations at a local scale (Ward et al., 2000). Therefore the longitudinal profile of the river is associated with two gradients towards the coast–a drier climate and more saline groundwater.

**Table 1:** Information about the Swakop River and its catchment.

Characteristic	Details
River length	460 km^1^
Catchment area	30,100 km^2^^1^
Annual mean temperature	18–22 °C (west–east)^2^^,^^3^
Annual total precipitation	0–475 mm/yr (west–east)^1^
Land tenure (by catchment area)	Private (89%), protected park land (8%), mining concessions (2%), communal land (1%)^1^^,^^4^
Population	180,000^1^^,^^5^
Dams	Sartorius Von Bach Dam: 48 mm^3^ capacity; 5 km^2^ surface area^6^
	(22°0′51″S 16°57′14″E)
	Swakoppoort Dam: 63 mm^3^ capacity; 8 km^2^ surface area^6^
	(22°12′45″S 16°31′35″E)
Riparian vegetation	Native trees: ana tree (*Faidherbia albida*), camel thorn (*Vachellia erioloba*), umbrella tree (*Vachellia tortilis*), fake ebony (*Euclea pseudebenus*), mustard bush (*Salvadora persica*), saltbush (*Tamarix usneoides*);
	Invasive trees: mesquite (*Prosopis* spp.)^1^

**Notes:**

1 Jacobson, Jacobson & Seely (1995).

2 Mendelsohn et al. (2009).

3 Large fluctuations occur at the daily, seasonal and annual scale.

4 Mining concessions have increased since this assessment as new uranium mining concessions have been granted in the western part of the catchment, within the Namib Naukluft National Park.

5 The urban and communal populations have no doubt grown since this assessment in the early 1990s, but the population living on farmland will not have substantially changed.

6 NamWater website, http://www.namwater.com.na.

Two large dams have been erected in the upper reaches of the river (Table 1). The first, built in 1970, was the Sartorius Von Bach Dam. This was followed in 1978 by the Swakoppoort Dam, 70 km downstream. As the second dam has no sluice gates to release water, the river below the dam is cut off from the flows upstream, except during exceptional flow conditions when the dam overtops. These dams have thus reduced the frequency and magnitude of flow events downstream (Ward et al., 2000; Jacobson & Jacobson, 2013). Prior to 1978 the river flowed nearly 90% of years and median annual flow approached 9 million m^3^; afterwards, the river flowed less than 50% of years and median annual flow reduced to 0.043 million m^3^ (as measured from a weir 15 km downstream of the Swakoppoort Dam between 1962–2005) (Douglas, 2014). Similarly, median annual river flow in the mid-reaches of the river is estimated to have reduced by 63% as a result of the two dams (Council for Scientific Industrial Research, 1997). The altered flow regime has reduced groundwater recharge, causing declines in groundwater levels downstream of the dams (Marx, 2009).

This paper focuses on the survival of the two most common woody species below the Swakoppoort Dam, the ana tree (*Faidherbia albida*) and mesquite (*Prosopis* spp.) (Table 1). The ana tree is an important component of the river system as it is the largest and most common native tree in the woodland community. In Namibia, ana trees are evergreen, grow to a maximum height of 20 m and are primarily found in rivers (Curtis & Mannheimer, 2005; Mannheimer & Curtis, 2010). The roots of ana trees access both soil and groundwater (Schachtschneider & February, 2010); with a maximum rooting depth of 40 m (Barnes & Fagg, 2003). The species uniquely produces pods during the dry season, thus acting as a valuable source of food and fodder for wild animals and livestock, and, due to its relative abundance and central position in the riverbed, it is a major contributor of organic matter to the ecosystem (Jacobson & Jacobson, 2013). Mesquite is similarly important as a locally abundant tree that provides shade and food to both wild animals and livestock (Smit, 2004). In Namibia, mesquite are evergreen, grow to a maximum height of 8 m and are primarily found in both rivers and plains (Curtis & Mannheimer, 2005; Mannheimer & Curtis, 2010). Mesquite access both soil and groundwater with a maximum rooting depth of 15–53 m (Nilsen et al., 1983; Stone & Kalisz, 1991; Schachtschneider & February, 2013). Mesquite was first introduced to Namibia in approximately 1912 (Smit, 2004). Of the six mesquite species successfully established, the most common along Namibia’s rivers are *P. glandulosa* var *torreyana*, *P. chilensis*, *P. velutina* and their hybrids (Smit, 2004). Due to frequent hybridization, this study does not attempt to differentiate between the species.

Patterns of human settlement in the catchment vary between three main types of land tenure: private, communal and park lands (Table 1). Private land generally constitutes a homestead with the surrounding land typically used for livestock and game farming, tourism, trophy hunting and/or small-scale crop production. Otjimbingwe, a 1,170 km^2^ reserve of communal land, straddles the Swakop River in the centre of the catchment. In 2000, as many as 8,000 people were estimated to live in the reserve, with between 800–2,000 people living in Otjimbingwe town and the remainder on the surrounding grazing land (Ward et al., 2000). Livelihood activities include livestock farming and some small-scale crop production (Ward et al., 1998). The main protected park land is the Namib-Naukluft National Park, created in its present form in 1979 and 49,768 km^2^ in size, located in the south-west of the catchment, where there are strict regulations on human activities. The Swakop River runs along, or in some places inside, the northern boundary of the park.

### River surveys

**Figure 1:**
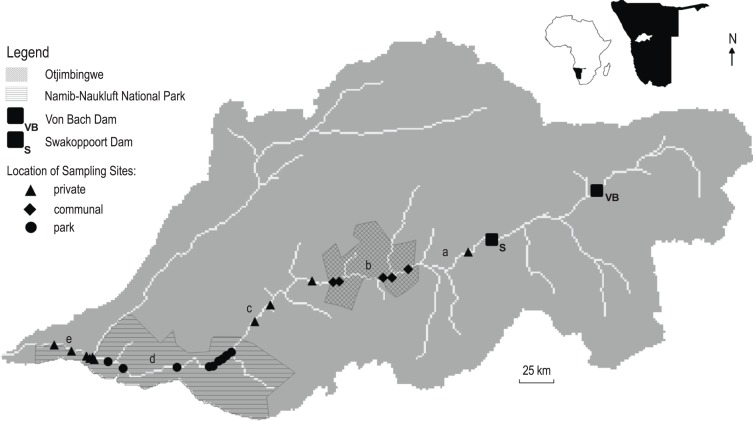
Maps of study area. Simplified map of the Swakop River catchment relative to its position in Namibia and Africa. Catchment map shows the distribution of land tenures and sampling regions. For illustrative purposes the river below the Swakoppoort Dam has been divided into five sampling areas (from east to west): zone a (private land with one sampling site), zone b (communal land with five sampling sites), zone c (private land with three sampling sites), zone d (park land with nine sampling sites) and zone e (private land with six sampling sites).

We surveyed 24 riverbed sites between 2 August and 24 October 2010; located between 22°26S 16°43E (17 km downriver of the Swakoppoort Dam) and 22°64S 14°75E (25 km upstream of the river mouth), covering more than 250 km of the river’s total 460 km length (see Fig. 1; Table S1). These sites were surveyed in a non-sequential order to minimize any temporal bias along the climate gradient associated with the river’s long profile. The 24 sites were distributed across three categories of land tenure with 42% of sites on private land, 21% on communal land and 37% on park land. This sampling effort is roughly proportional to the coverage of the land tenures along the river’s borders within the study area: 55% private, 15% communal and 30% park (Fig. 1). A permit for this research was obtained from the Republic of Namibia’s Ministry of Environment and Tourism (Permit Number 1517/2010).

At each site, we placed four 20 m wide transects, oriented perpendicular to the river course and of variable length as determined by the width of the riverbed at that location. Each site was located at a borehole, and the transects placed in such a way that the two central transects were placed adjacent to each other, with their shared boundary running directly through the centre of the borehole, and the two remaining transects placed 250 m upriver and downriver of the central transects. At one site, three boreholes were located in close proximity (< 1 km), so transects were only laid over the boreholes, generating six transects at that site. We divided each transect into a series of consecutive 20 × 20 m quadrats, for a median number of 20 quadrats per transect (range: 9–52).

Four individuals collected the data and to maintain consistency the role of each person was kept constant throughout data collection. For each tree (minimum diameter of 5 cm) we collected data on: species, location on transect (*i.e.* which quadrat), diameter of trunk above the buttress swelling and percentage canopy dieback. A diameter tape was used to measure tree diameter, but if the trunk was inaccessible due to impenetrable vegetation it was estimated with a ruler (10% of cases) or visually (24% of cases). Two researchers independently visually estimated percentage canopy dieback (*i.e.* 0–100% of the canopy comprising of dead material) before reaching a consensus. We collected data on living and dead (100% canopy death; standing and fallen) trees. We checked that any fallen trees included in the study had once been standing within the quadrat and were not washed-in from upstream (by observing orientation and/or the presence of a stump of the same size/species). We also measured topography to assess both the elevation above and the cross-sectional distance from the lowest point in the transect; topography was recorded by using a clinometer to measure slope gradients and a combination of quadrat lengths and paces to determine distance (Article S1).

### Quantifying dieback

Due to the need to include dead trees in the analysis, and the large number of dead trees in the sample, we used a binomial response variable to quantify dieback. Because canopy dieback is an adaptive response to drought for desert trees (Scott, Shafroth & Auble, 1999; Schachtschneider, 2010), we consider low or moderate levels of dieback to be a natural pattern from which healthy trees may recover. However, high canopy dieback is likely to be strongly associated with tree mortality. On this basis, we classify ‘high’ dieback as all those trees with ≥ 75% dieback, including dead trees (100% dieback). All other trees, including those with completely healthy canopies (0% dieback), were classed as ‘low’ dieback. Both standing and fallen dead trees were included in the analysis to reduce any bias introduced if only standing dead trees were included (the latter would underestimate the dieback of those species which tend to fall more quickly after death). The age of dead trees in this sample is difficult to estimate with precision, but the low rates of woody decomposition in arid environments (Vogel, 2003) suggest that our sample is likely to include all trees that have died since damming in 1978, with the exception of those that may have been swept away in the rare and localised flows that have occurred following damming (Jacobson, Jacobson & Seely, 1995; Douglas, 2014). In contrast, few trees that died prior to damming are likely to have been present as the natural seasonal flows at this time would have washed such trees away (Auala et al., 2013).

### Statistical analysis

We analysed tree dieback at the quadrat level using general linear mixed models with a binomial error structure and logit link function, implemented in Rv3.3.0 (R Development Core Team, 2016) using the ‘glmer’ function in the lme4.0 package (Bates et al., 2015). Our response variable, median dieback, categorised quadrats as ‘low’ dieback (< 75%) or ‘high’ dieback (≥ 75%) according to the median value of dieback across all trees in that quadrat. Dieback was analysed for the ana tree (n = 268 quadrats) and mesquite (n = 437 quadrats) in separate models.

The full models contained five or six fixed effects (depending on the taxon) across two spatial scales, summarized as follows. At the quadrat level there were three fixed effects. These comprised two measures of local topography, namely the horizontal *distance* to the lowest point across the transect (m) and the *elevation* above the lowest point in the transect (m) (Article S1), and, for the ana tree model only, the relative abundance of *mesquite* (using the total basal area of all mesquite in the quadrat divided by the total basal area of all trees in the quadrat). At the site level there were also three fixed effects. These comprised *land tenure* (a categorical variable with three levels: private, n = 9 sites; communal, n = 5 sites; and park, n = 8 sites), *dryness* (the ratio of potential evapotranspiration to precipitation) representing the climatic gradient associated with the longitudinal profile of the river (Article S2), and *tributary*. *Tributary* was a categorical variable of two levels: the presence/absence of upstream tributary flow (n = 12 sites in each case), calculated from runoff modelled through ≥ third Strahler stream-order tributaries within 5 km upstream of each site (Article S3). A large number of interactions could also be envisaged between these six fixed effects, but our ability to confidently test for such interactions was limited by sample size. Consequently, we focused solely on main effects.

To compare differences in the magnitude of effect between variables, we standardised all the continuous fixed effects to have a mean of zero and a standard deviation of one. We also included two random effects to control for any possible non-independence resulting from the sampling design: *transect* (multiple at each site) (n = 98) nested within *site* (n = 24). Models were not assessed for overdispersion because overdispersion cannot be quantified for binary models (Harrison, 2015). We assessed the collinearity between the fixed effects (Figs. S1 and S2). Potential collinearity between the continuous fixed effects was assessed through correlation ellipses and Pearson’s correlations. Any collinearity between the categorical fixed effects was assessed with a correlation matrix. In all but one case, no evidence of collinearity was found, with all correlation coefficients < 0.3, substantially lower than the standard |r| > 0.7 threshold (Dormann et al., 2013). The only exception was a strong relationship between our two measures of topography, distance and elevation, for the ana tree (r = 0.8). As a result these two predictors were never included together in the same model.

We used an information-theoretic approach for model selection (Burnham & Anderson, 2002), in which we considered all possible combinations of fixed effects in our candidate models (with the exception of elevation and distance: see above). The candidate model sets thus consisted of 48 and 24 models for the ana tree and mesquite, respectively. We used the R package ‘MuMIn’ (Bartoń, 2016) to rank the models according to Akaike’s information criterion (AIC). We considered all models within Δ6 AIC units of the best-supported model as competitive (Richards, 2008). As AIC tends to select unnecessarily complex models (Richards, 2008; Arnold, 2010), we then applied the ‘nesting rule’ to the Δ6 top model set, where we removed models that were more complex versions of simpler models with better AIC support (Richards, 2008). Where more than one model remained in the top model set after applying the nesting rule, we performed model averaging to calculate the parameter estimates and standard errors of a final composite model using the ‘model.avg’ function in the MuMIn package. We performed full-coefficient (‘zeroes’) modelling, where parameter estimates were set to zero in models in the top model set from which they were absent so as to fully propagate uncertainty in parameter effect sizes and standard errors (Grueber et al., 2011). We used confidence intervals to assess the strength of the fixed effects in the models with substantial support (du Prel et al., 2009). Only the fixed effects whose confidence intervals did not cross zero were treated as having support; if their confidence interval crossed zero, the result was considered inconclusive.

## Results

Individual tree dieback ranged from zero to completely dead (100% dieback) in our sample. The native ana tree (n = 750 individuals) experienced the most dieback, with 51% of individuals completely dead. Living individuals had a median canopy dieback of 18% (range: 0–90%). For the invasive mesquite (n = 1,160 individuals), 26% of individuals were completely dead. Living individuals had 10% median canopy dieback (range: 0–90%).

### Ana trees

**Figure 2:**
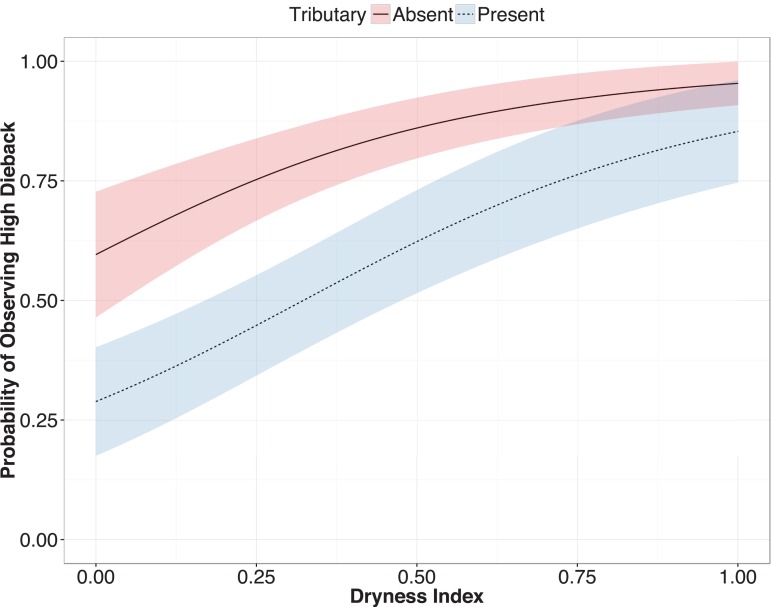
Predicted probability of observing high ana tree dieback in relation to upstream tributary presence and dryness. Dashed lines: tributary present; solid line: no tributary present. Fitted lines are mean predicted probability from a binomial mixed effects model. Shaded areas are +/− SE. The model predicts a higher probability of observing high dieback where upstream tributaries are absent and the climate is drier.

The top model set examining factors affecting tree dieback in the ana tree population contained three models, of which only one was retained following application of the nesting rule (Table S2). The retained model provided support for three of our five hypotheses. High tree dieback was associated with a drier climate (H2), the absence of runoff from nearby upstream tributaries (H3) (Fig. 2) and an increased abundance of invasive tree species (H5) (Table 2). We also found mixed support for our hypothesis of increased dieback resulting from human settlement (H4): specifically, dieback was highest on private land, as predicted, but lowest in communal land, contrary to prediction. The effects of park land could not be reliably distinguished from those of private land due to confidence intervals that crossed zero. There was no effect of distance from (H1.1), or elevation above (H1.2), the channel’s lowest point.

**Table 2:** Model coefficients, standard errors and 95% confidence intervals for binomial mixed effects models investigating the factors influencing probability of observing high dieback in ana trees and mesquite. Ana tree coefficients are derived from the best supported AIC model (*F. albida*, n = 268). Mesquite coefficients are model-averaged coefficients from the three best-supported models (*Prosopis* species, n = 437). All coefficients are in logits. Low dieback (0–74%) is coded 0 and high dieback (75–100%) coded as 1; all continuous fixed effects were standardized (mean of zero, standard deviation of one).

Species	Parameter		Estimate	SE	95% CI	
					Lower	Upper
Ana tree^a^	Intercept		1.03	0.33	0.40	1.7
	Tributary^b^		−1.16	0.33	−1.84	−0.52*
	Dryness		0.55	0.19	0.19	0.96*
	Land tenure^c^	Communal	−2.55	0.48	−3.55	−1.64*
		Park	−0.44	0.34	−1.11	0.21
	Mesquite		1.78	0.59	0.68	3.02*
Mesquite^d^	Intercept		−1.79	0.43	−2.63	−0.95
	Elevation		0.19	0.19	−0.18	0.57
	Land tenure^c^	Communal	−2.82	1.39	−5.56	−0.07*
		Park	−0.29	0.64	−1.64	0.97

**Notes:**

a Ana tree estimates are from the best supported AIC model.

b Reference category for Tributary is ‘No Tributary.’

c Reference category for Land Tenure is ‘Private.’

d Mesquite is a composite model averaged from three models.

* Support for parameter (*i.e.* confidence interval does not cross zero).

### Mesquite trees

In the case of the determinants of dieback in the mesquite population, the top model set contained 14 models, of which three remained after application of the nesting rule (Table S3). We found mixed support for our human settlement hypothesis (H4) (Table 2). Specifically, like the ana tree, dieback was highest on private land and lowest in communal land, with park land occupying an equivocal position. The effects on tree dieback of elevation above the channel’s lowest point (H1.2) were inconclusive. There was no effect of distance from the channel’s lowest point (H1.1), a drier climate (H2) or tributary flow (H3) on mesquite dieback.

## Discussion

Our study has explored the processes that drive patterns of tree dieback along a large ephemeral dryland river subject to disrupted river flow from damming, human settlement and alien tree invasion. In this river system, the Swakop River in Namibia, we found an exceptionally high level of dieback in the native ana tree, with 51% of individuals completely dead. This compares with only 4% mortality in an undisturbed population on the Kuiseb River, another large ephemeral river whose catchment neighbours the Swakop but which is undammed and contains few mesquite trees (Auala et al., 2013). The high mortality we observed is also nearly double that reported for an intermittent river in South Africa with small dams along its tributaries, where 29% of ana trees were reported dead (O’Connor, 2001). While it is possible that dead trees may remain longer in the Swakop than in undammed river systems, where natural flows wash them away, it is unlikely that this alone could account for the extreme levels of mortality observed. Such an explanation would also fail to account for the high estimates of canopy dieback in the living trees, which are very likely conservative since ana trees can drop dead branches during times of stress (Barnes & Fagg, 2003).

The invasive mesquite also showed a relatively high level of decline. Although we have been unable to locate natural mortality data from other populations, our observation of 26% of individuals dead suggests that the same processes affecting the ana tree are also affecting the mesquite. It also suggests that the decline of ana trees is unlikely to reflect the influence of mesquite alone. However, we were only able to identify one factor associated with mesquite dieback: a land tenure effect that, as in the ana tree, most likely represents an artificial bias (see our discussion of land tenure effects two paragraphs below). Our inability to identify a comparable set of predictors for mesquite and ana tree dieback does not mean that their respective declines do not share a common cause. Rather, it suggests that it is more difficult to detect in the former, which has wider environmental tolerances and drought avoidance mechanisms (Smit, 2004; Schachtschneider & February, 2013), while the latter is more vulnerable to drought and hydrological change (Ward & Breen, 1983; O’Connor, 2001; Muller, 2003; O’Connor, 2010; Schachtschneider, 2010). The remainder of this Discussion therefore focuses on the ana tree.

Our analyses of the potential drivers of ana tree dieback found strong support for two of the three hypotheses concerning flow effects, namely dryness and upstream tributary presence. This suggests that the reduction in the frequency and magnitude of flows on the Swakop River associated with the building of large dams in its upper catchment in the 1970s has had a major impact on the native ana tree. Two further predictors of ana tree dieback were land tenure and mesquite abundance. However, the interpretation of these latter two predictors is less straightforward.

In the first case, although tree dieback was highest on private land (as predicted), it was lowest on communal land (contrary to prediction), and the difference between park and private land was equivocal. Livestock is present on both communal and private lands on the Swakop River, and may reduce sapling survival (Moser, 2006), but browsing impacts are unlikely to influence the patterns of dieback reported here since only larger trees were surveyed. Moreover, browsing impacts would lead to lower dieback in park land, which was not observed. Similarly, recent or current water abstraction was observed at 100% (5/5) of sampling sites within the communal land, 78% (7/9) of private sites, and 13% (1/8) of the park sites, suggesting that these patterns cannot be explained by the presence/absence of water abstraction either. However, presence/absence is only a crude measure of water abstraction, since abstraction rates and periods vary over time, especially relative to local groundwater availability. It therefore remains possible that water abstraction does contribute to tree dieback along the Swakop (Muller, 1985; O’Connor, 2010; Wassenaar, Shuuya & Mbura, 2014). Nevertheless, water abstraction is unlikely to explain the low dieback on communal land. An alternative possibility might be that a lower tree biomass in communal areas, due to heavier livestock browsing and human exploitation, has reduced intraspecific competition. However, communal land had the highest ana tree biomass of all land tenures (Douglas, 2014). A more likely explanation is that the low dieback reflects the removal of dead limbs and trees by local people for use as fuel, fencing and/or building materials (Barnes & Fagg, 2003; Moser, 2006), which deflates the apparent dieback levels. Thus, the lower dieback in communal areas, relative to private land and park land, most likely reflects an artefact of dead wood collection rather than a genuine relationship between this land tenure and improved tree health.

In the second case, ana tree dieback was more severe in areas of higher mesquite abundance, as predicted. However, while this finding supports our hypothesis it should be interpreted with caution, because we cannot distinguish between whether the mesquite is the cause of dieback or simply establishing itself where native trees are already dying from other threats, *i.e.* whether mesquite is a ‘driver’ or ‘passenger’ of environmental change (MacDougall & Turkington, 2005). That mesquite is more likely to be a driver is supported by recent research on a South African ephemeral river demonstrating that the native camel thorn (*Vachellia erioloba*) experiences higher water stress and greater dieback in the presence of mesquite (Schachtschneider & February, 2013). Further research is required on the Swakop populations; stable isotope analysis and dendrochronology could be particularly useful in establishing the timing of tree death in relation to dam building and mesquite arrival.

An impact of damming on native tree populations was supported by two of three flow-related predictors of dieback, namely dryness and tributary flow. There was no effect of the third predictor, position in riverbed, perhaps because the benefits and costs of a particular position are inconsistent over time as the topography of the river surface changes due to fluctuations in local flow volume. In the case of dryness, ana trees showed higher dieback in drier areas, consistent with the hypothesis that trees with greater climatic stress are more vulnerable to post-damming reductions in surface flows and groundwater levels. Because drier areas also experience higher groundwater salinity in the Swakop system (Gevers & Westhuyzen, 1937), this finding may reflect a combination of local stressors. In the case of tributary flow, ana trees showed higher survival when local flows from nearby upstream tributaries were present (Fig. 2). Such an effect could reflect a variety of local flow benefits, including the physical action of water (scouring), deposition of nutrient-rich sediments, recharge of groundwater, or some combination thereof.

One limitation in our study has been our inability to explore the effects of potential interactions between our five predictors due to the relatively small number of sites we were able to survey (n = 24) and the potentially large number of interactions. For instance, we might expect the protective effects of tributaries to be more accentuated in the drier stretches of the Swakop, or for the deleterious effects of mesquite to be greater where tributary flow is absent. Nevertheless, our identification of the direct effects of our five predictors on tree dieback provides a starting point for future research that we hope will explore such potential interactions, as well as elucidate in more detail the precise mechanisms that are involved in each case.

## Conclusions

Although the precise mechanism remains to be identified, our results highlight the valuable role that tributaries seem to play in protecting riparian woodlands in dammed ephemeral rivers, in line with their role in protecting ecosystem structure and function in dammed perennial rivers (Rice et al., 2008). The management implications of beneficial tributary flow in dammed ephemeral rivers are manifold. One obvious solution to the problem of riparian tree mortality from impounded flows on the Swakop River would be the controlled release of water to simulate such flows. However, a recent study of controlled releases from a dam on another of Namibia’s ephemeral rivers found these to be ineffective, as the amount of water that could be released without jeopardising urban water supply was too small to mimic natural flows and, unlike natural flows, was devoid of silt and organic matter (Jacobson & Jacobson, 2013). Moreover, the Swakoppoort Dam has no sluice gates.

Under these conditions, the protection of tributary flow is likely to be the most effective way of maintaining riparian tree populations, at least in those areas where tributaries are present. Although the main tributary of the Swakop (the Khan) is not dammed, there are many smaller tributaries that are vulnerable to damming by local people. Our ability to detect a tributary effect suggests their flows have been largely maintained up until now. Nevertheless, a recent study identified 1,220 ‘farm’ dams in the catchment, with 17% of these dams possessing a storage capacity greater than 20,000 m^3^ (range: 44–267,935 m^3^) (Parker, 2012). Current legislation bans activities which impede flow in watercourses without prior approval (Government of Namibia, 2013). However, prior to The Water Resources Management Act, approval was only required for the construction of dams larger than 20,000 m^3^; moreover, larger dams were often constructed illegally (Jacobson, Jacobson & Seely, 1995). Although farm dams provide much needed water in the dry season, they withhold flow to the detriment of downstream users, and their ecological impacts are unknown. The new Water Act shows that the importance of river flow is recognised in Namibia. Our findings support this legislation and the need for a more active approach to tributary management on Namibia’s ephemeral rivers.

## Supplemental Information

10.7717/peerj.2622/supp-1 Supplemental Information 1 Survey locations. Location of river survey sampling sites in order from east to west (i.e. downstream) along the Swakop River. The corresponding zones of each sampling site are shown in Fig. 1.

10.7717/peerj.2622/supp-2 Supplemental Information 2 Topography (Elevation and Distance).

10.7717/peerj.2622/supp-3 Supplemental Information 3 Dryness Index.

10.7717/peerj.2622/supp-4 Supplemental Information 4 Tributary Metric.

10.7717/peerj.2622/supp-5 Supplemental Information 5 Relationships between the continuous fixed effects used in the ana tree (*F. albida*) dieback analysis. ‘elev’ = elevation; ‘dist’ = distance; ‘aridindex’ = dryness index. Correlation ellipses are shown in the upper diagonal and Pearson’s r correlation coefficients are shown in the lower diagonal. Correlation of categorical fixed effects was assessed using the global model fixed effects correlation matrix, where no strong correlations were detected (all values < 0.26 and > −0.22).

10.7717/peerj.2622/supp-6 Supplemental Information 6 Relationships between the continous fixed effects used in the mesquite (*Prosopis* spp.) dieback analysis. ‘elev’ = elevation; ‘dist’ = distance; ‘aridindex’ = dryness index. Correlation ellipses are shown in the upper diagonal and Pearson’s r correlation coefficients are shown in the lower diagonal. Correlation of categorical fixed effects was assessed using the global model fixed effects correlation matrix, where no strong correlations were detected (all values < 0.36 and > −0.14).

10.7717/peerj.2622/supp-7 Supplemental Information 7 Model selection results for ana tree dieback.

10.7717/peerj.2622/supp-8 Supplemental Information 8 Model selection results for mesquite dieback analysis.

10.7717/peerj.2622/supp-9 Supplemental Information 9 Raw data for ana tree dieback analysis. ‘site’: ID of site; ‘transect’: ID of transect within each site; ‘quadrat’: ID of quadrat within each transect; ‘median.perc75’: median dieback per quadrat 1 = high dieback ≥ 75%, 2 = low dieback < 70% (binary); landtenure: 1 = private, 2 = communal, 3 = park (nominal); trib: 1 = tributary, 2 = no tributary (nominal); mesquite: proportion of mesquite; elev.s: standardised elevation; dist.s: standardised distance; arid.s: standardised dryness.

10.7717/peerj.2622/supp-10 Supplemental Information 10 Raw data for mesquite dieback analysis. ‘site’: ID of site; ‘transect’: ID of transect within each site; ‘quadrat’: ID of quadrat within each transect; ‘median.perc75’: median dieback per quadrat 1 = high dieback ≥ 75%, 2 = low dieback < 70% (binary); landtenure: 1 = private, 2 = communal, 3 = park (nominal); trib: 1 = tributary, 2 = no tributary (nominal); elev.s: standardised elevation; dist.s: standardised distance; arid.s: standardised dryness.

## Additional Information and Declarations

### Field Study Permissions

The following information was supplied relating to field study approvals (i.e., approving body and any reference numbers): A permit for this research was obtained from the Republic of Namibia’s Ministry of Environment and Tourism (Permit Number 1517/2010).

### Data Deposition

The following information was supplied regarding data availability: The raw data has been supplied as Supplemental Dataset Files.
