# Supplementary material for: Widespread dieback of riparian trees on a dammed ephemeral river and evidence of local mitigation by tributary flows
Source: PeerJ. 2016 Oct 27;4:e2622. doi: 10.7717/peerj.2622 (PMC5088575; doi:10.7717/peerj.2622)
Supplement: Supplemental Information 1 — Location of river survey sampling sites in order from east to west (i.e. downstream) along the Swakop River. The corresponding zones of each sampling site are shown in Fig. 1. [file peerj-04-2622-s001.pdf]

Site	Latitude	Longitude	Zone
A	-22.262803°	16.430612°	a
B	-22.334851°	16.185031°	b
C	-22.368852°	16.120811°	b
D	-22.368847°	16.083167°	b
E	-22.384594°	15.905126°	b
F	-22.386272°	15.879608°	b
G	-22.381614°	15.795242°	c
H	-22.478235°	15.623867°	c
I	-22.545853°	15.561153°	c
J	-22.671761°	15.467223°	d
K	-22.684881°	15.446948°	d
L	-22.697276°	15.429669°	d
M	-22.707238°	15.414449°	d
N	-22.727822°	15.392948°	d
O	-22.729756°	15.377145°	d
P	-22.732351°	15.244313°	d
Q	-22.735489°	15.026310°	d
R	-22.709827°	14.964951°	d
S	-22.699547°	14.908687°	e
T	-22.692308°	14.901570°	e
U	-22.695429°	14.888835°	e
V	-22.686849°	14.875461°	e
W	-22.665997°	14.816335°	e
X	-22.642124°	14.746183°	e
