# Supplementary material for: Widespread dieback of riparian trees on a dammed ephemeral river and evidence of local mitigation by tributary flows
Source: PeerJ. 2016 Oct 27;4:e2622. doi: 10.7717/peerj.2622 (PMC5088575; doi:10.7717/peerj.2622)
Supplement: Supplemental Information 2 [file peerj-04-2622-s002.pdf]

Supplementary Article S1 - Topography (Elevation and Distance)

Local topography, *i.e.*, the position of a quadrat on the cross-section of the river, was assessed according to both the elevation above the lowest point in the river channel, and the distance from the lowest point in the river channel. To facilitate data collection, this cross-sectional topography was measured using zones which represented areas of assumed homogenous topography between user-defined breaks of slope. At the start of each zone, the angle of incline or decline was measured with a clinometer. The length of the zones was measured using a combination of quadrats and paces. As quadrats were 20m in length, the length of a zone could be quickly calculated by adding the number of whole quadrats contained within each 'zone'. However, if a zone covered only part of a quadrat, paces were used to identify the length of that zone. The length of a zone was determined from either paces, quadrats or both, and the final length calculated in meters. A single quadrat could be in more than one zone depending on the location of the breaks of slope identified. During data entry and processing, all the zones were broken into their constituent quadrats and their individual length contributions (hereafter: mini-zone).

The length measurements were made along the surface of the riverbed (*i.e.*, its cross-sectional profile). However as we were interested in the height and Euclidean horizontal distance at each point, the length measurements were converted into vertical heights (elevation) and horizontal distances (distance), respectively. The elevation and distance measurements were taken in relation to the lowest point in the river because it was assumed that this would be the location where the river would normally flow. The horizontal distances and vertical heights from the start and finish of each mini-zone (hereafter: data points) were calculated using trigonometry.

As the hypotenuse and corresponding angle were known, the cosine and sine functions were used to calculate the elevation (opposite) and distance (adjacent) measurements, respectively.

Starting with the left hand side of the transect when facing upstream (*i.e.*, the north bank; Quadrat A in Fig. A), we added the horizontal distances together so that the cumulative distance of each data point from the left side of the transect was known. We used the same approach for the (positive and negative) change in height data. From the height measurements we identified the lowest point along the transect and then calculated the height difference between this lowest point and all other data points. This resulted in the lowest point in the transect having a height of zero and all other quadrats a positive height measurement. Further details are provided below.

Distance

To find the distance from the lowest point in the riverbed, we first subtracted the horizontal distance of the lowest data point in the riverbed from every other data point. This calculation provided us with the distances from the lowest point in the riverbed to the start/finish of each quadrat (Q_{end} in Fig. A). However we wanted to calculate the distance measurements from the centre of the quadrat, as opposed to the start or end of a quadrat (*i.e.* Distance in Fig. A). Therefore depending on the quadrat's location in the transect, 10 m (half the length of a quadrat) was either added or subtracted to the distance measurement at the end of the quadrat. Therefore if the quadrat was located to the left of the lowest quadrat then 10 m was added because the centre of the quadrat was in fact 10 m further away than its edge (*i.e.* Quadrats A – D in Fig. A). In contrast, if the quadrat was located to the right of the lowest point, 10 m was subtracted from

the quadrat distance as the edge of the quadrat was in fact 10 m further away from the lowest point than the quadrat's centre *(i.e.* Quadrats E – H in Fig. A).

Elevation

Each quadrat had at least two height values as each zone had a start and finish elevation, and there could be more than one zone in each quadrat. Multiple steps were therefore required to obtain a representative elevation for each quadrat. Briefly, the area of each 'mini-zone' was calculated using the formula for a trapezoid as both height values and the length of the mini-zone were known (H_{start}, h_{end} and length in Fig. A). If more than one mini-zone was within a quadrat, the relevant mini- zones were then summed together. Knowing the area of the quadrat (*i.e.* the area of the mini-zone(s)) and the horizontal length of the quadrat, a representative height for each quadrat was determined (*i.e.* Elevation in Fig. A)

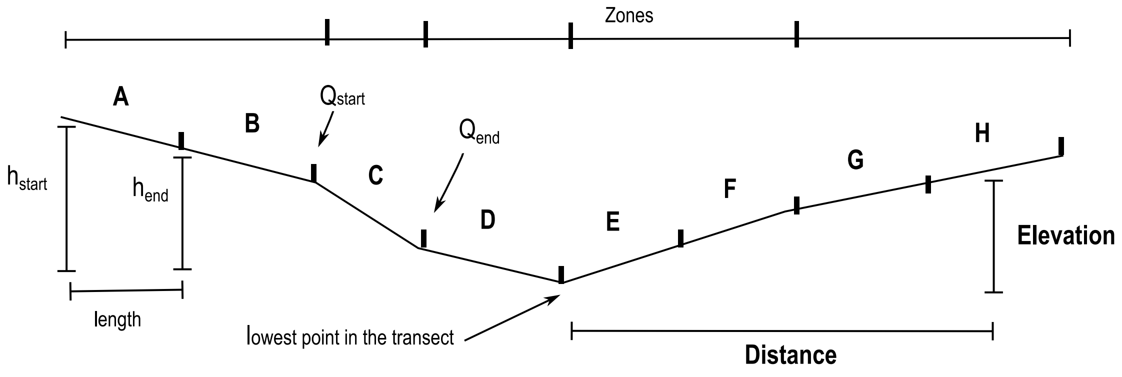

Figure A. Topography measurements for each quadrat. Distance represents the horizontal distance between the centre of a quadrat and the lowest point along the river's cross-sectional profile. Elevation represents the mean height of the quadrat above the lowest point along the river's cross-sectional profile. The capital letters in the figure illustrate the location of the square quadrats (20 m in length). Zones represent areas of homogenous topography. For ease of illustration, in this figure the boundaries of quadrats are the same as 'mini-zones' described in the above text.
