# Supplementary material for: Widespread dieback of riparian trees on a dammed ephemeral river and evidence of local mitigation by tributary flows
Source: PeerJ. 2016 Oct 27;4:e2622. doi: 10.7717/peerj.2622 (PMC5088575; doi:10.7717/peerj.2622)
Supplement: Supplemental Information 3 [file peerj-04-2622-s003.pdf]

Supplemental Article S2 - Dryness Index

In order to assess the climate of each sampling site, we created a dryness index based on dividing potential evapotranspiration (PET) by precipitation (P). Thus, wet areas have a low dryness index and arid areas have a high dryness index. Existing raster maps were used in the dryness index calculation, which was performed in PCRaster (PCRaster Research and Development Team, 2009). The resulting file was then exported to QGIS (Quantum GIS Development Team, 2012) and grid cell values were extracted for each sampling site. The maps used in the calculations covered a 10 degree extent with a 1 km^2 spatial resolution and were obtained from the WaterWorld Policy Support System (Mulligan, 2013). The precipitation map (Hijmans et al., 2005), available as an input map in WaterWorld, is a global dataset largely based on mean monthly precipitation data from monitoring stations between the dates of 1950-2000. Hijmans et al. (2005) extrapolated the station data using tension splines with elevation as a co-variable. The PET map was calculated in WaterWorld (v. 2.3) (Mulligan, 2012) and subsequently downloaded for use in this analysis. WaterWorld calculates PET based on a Penman-type approach (Penman, 1948) which is based on the energy available (*R*, *i.e.*, the surface net radiation) and the slope of the saturation vapour pressure versus temperature relationship at the ambient air temperature (Δ) and the psychrometric constant (Mulligan, 2013) (Eq. (1)). Because a few grid cells within the 10 degree extent, but not within the catchment, had PET values of zero (due to missing data), a small number (0.000001) was added to each grid cell to avoid division by zero (using PCRaster).

$$PET = (\Delta / (\Delta + \Upsilon)) \times R \qquad (1)$$

where:
PET = potential evapotranspiration (W/m^2)
Δ = slope of the saturation vapour pressure curve ($kPa/^oC$)

Υ = the psychrometric constant (0.666 kPa /^{o}C)
R = Net radiation receipt (W/m^2)

Within the 10 degree tile used in WaterWorld, the precipitation map is based on 161 stations and the temperature map based on 50 stations. Of those stations, 14 precipitation and 7 temperature monitoring stations are within the Swakop catchment (~30,000 km^2 area). The pattern of decreasing precipitation westward along the catchment shown in the gridded data matches the precipitation patterns shown in the Namibian literature (*e.g.,* Jacobson et al., 1995; Mendelsohn et al., 2009). The precipitation dataset used was selected over an alternate global precipitation dataset (TRMM 2B31 data accessed through WaterWorld) because the former better matched the Namibian data. The temperature data (Hijmans et al., 2005) adheres to the broad pattern shown in the *Atlas of Namibia* (Mendelsohn et al., 2009).

References

Hijmans R J,Cameron SE, Parra JL, Jones PG, Jarvis A. 2005. Very high resolution interpolated climate surfaces for global land areas. *International Journal of Climatology* 25: 1965–1978.

Jacobson P, Jacobson K, Seely M. 1995. *Ephemeral rivers and their catchments: sustaining people and development in western Namibia*. Desert Research Foundation of Namibia, Windhoek, Namibia.

Mendelsohn J, Jarvis A, Roberts C, Robertson T. 2009. *Atlas of Namibia: a portrait of the land and its people*. Sunbird Publishers, Windhoek, Namibia.

Mulligan M. 2012. WaterWorld Policy Support System version 2.3, Available from: http://www.policysupport.org/waterworld

Mulligan M. 2013. WaterWorld: a self-parameterising, physically based model for application in data-poor but problem-rich environments globally. *Hydrological Research* 44: 748.

PCRaster R&D Team. 2009. PCRaster software, Available from: http://pcraster.geo.uu.nl/.

Penman H L. 1948. Natural evaporation from open water, bare soil and grass. *Proceedingsof the RoyalSociety of London* A: 120–145.

Quantum GIS Development Team. 2012. Quantum Geographic Information System Software, version 1.7, Available from: http://qgis.osgeo.org
