# Supplementary material for: Widespread dieback of riparian trees on a dammed ephemeral river and evidence of local mitigation by tributary flows
Source: PeerJ. 2016 Oct 27;4:e2622. doi: 10.7717/peerj.2622 (PMC5088575; doi:10.7717/peerj.2622)
Supplement: Supplemental Information 4 [file peerj-04-2622-s004.pdf]

Supplementary Article S3 - Tributary Metric

The tributary metric is an indicator of tributary runoff experienced at each sampling site, where runoff was calculated in WaterWorld Policy Support System (v 2.7) (Mulligan, 2013a). Only tributaries with a Strahler stream order greater than or equal to three were included in the metric, as second-order streams were not easily identifiable using Google Earth imagery (Google Inc, 2013). A scatter plot of stream order versus runoff, for a random sample of points within the catchment, shows a clear relationship between stream order and runoff. Neither first nor second order streams had a total annual runoff greater than 0.0 m^3/s (to one decimal place). In contrast, third order streams had a mean annual runoff of 0.2 m^3/s (min: 0.0 m^3/s; max: 0.8 m^3/s), supporting our decision to use this order as a minimum for streams of significance. The total runoff contributed by tributaries up to 5 km upstream of each sampling site was calculated. A 5 km distance was adopted as it represented the smallest distance we could robustly use given the coarseness of the input data (1 km^2 resolution) and associated uncertainties in the digital elevation model used to derive the stream flow network. The 5 km distance upstream (calculated by following the path of the riverbed) was measured in Google Earth. The Google Earth imagery was then overlaid with a stream order (≥3) map and the tributaries within 5 km of each sampling site were identified. The runoff values for the identified tributaries were then queried in WaterWorld and their runoff rates summed.

Given the uncertainties with tributary runoff, tributary flow was quantified as a binary rather than continuous variable. The key uncertainty was the difficulty in accommodating the spatial variation of tributary influence within the 5 km area, *e.g.*, a tributary 4 km upstream will likely have less influence on a sampling site than a similarly sized tributary 1 km upstream.

Therefore the total runoff values within 5 km upstream of each site were collapsed into a binary variable – ‘no tributary flow’ (runoff< 1 m^3/s) and ‘tributary flow’ (runoff$\geq 1m^3/s$). The 1 m^3/s threshold was selected because it allowed for a relatively equal sample size between categories.

In WaterWorld, runoff is estimated by accumulating annual total water balance (rainfall + fog + snowmelt inputs - actual evapotranspiration, Eq. (1)) along a local drainage direction map (Mulligan, 2013b). The local drainage direction map used in WaterWorld is HydroSHEDS (Lehner et al., 2008).

$$WB = (P + F + S) - AE \quad (1)$$

where:
WB=water balance (mm/yr)
P = precipitation (mm/yr)
F = fog interception (mm/yr)
S = water input from snowmelt (mm/yr)
AE = actual evaporation (mm/yr)

References

Google Inc. 2013.Google Earth version 7. Available from: http://earth.google.com

Lehner B, Verdin K, Jarvis A. 2008. New global hydrography derived from spaceborne elevation data. Eos, *Transactions of American Geophysical Union* 89: 93–94.

Mulligan M. 2013a. WaterWorld Policy Support System version 2.9, Available from: http://www.policysupport.org/waterworld

Mulligan M. 2013b. WaterWorld: a self-parameterising, physically based model for application in data-poor but problem-rich environments globally. *Hydrological Research* 44: 748.
