# Supplementary figures and images for: Widespread dieback of riparian trees on a dammed ephemeral river and evidence of local mitigation by tributary flows
Source: PeerJ. 2016 Oct 27;4:e2622. doi: 10.7717/peerj.2622 (PMC5088575; doi:10.7717/peerj.2622)

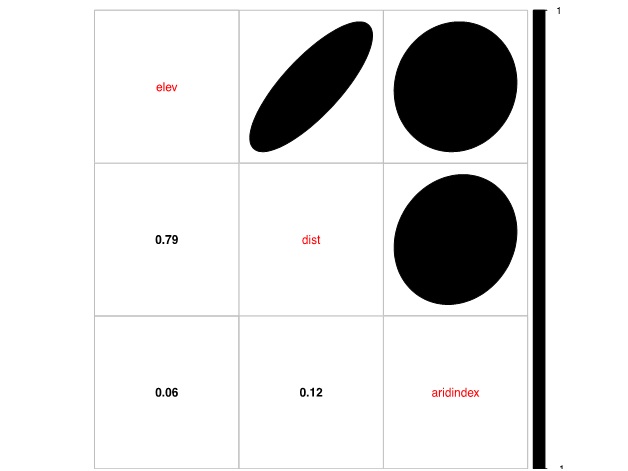

Supplement: Supplemental Information 5 — ‘elev’ = elevation; ‘dist’ = distance; ‘aridindex’ = dryness index. Correlation ellipses are shown in the upper diagonal and Pearson’s r correlation coefficients are shown in the lower diagonal. Correlation of categorical fixed effects was assessed using the global model fixed effects correlation matrix, where no strong correlations were detected (all values < 0.26 and > −0.22). [file peerj-04-2622-s005.jpg]

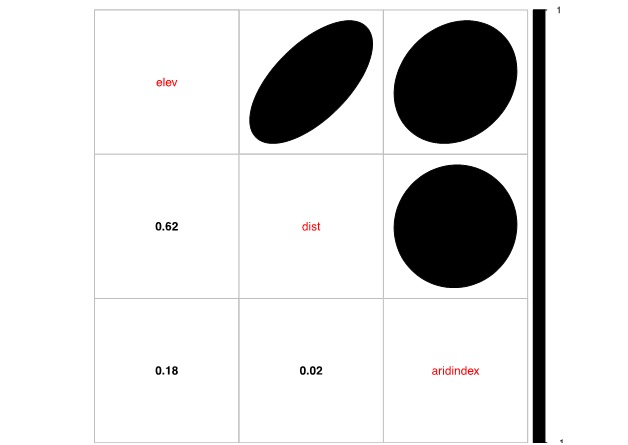

Supplement: Supplemental Information 6 — ‘elev’ = elevation; ‘dist’ = distance; ‘aridindex’ = dryness index. Correlation ellipses are shown in the upper diagonal and Pearson’s r correlation coefficients are shown in the lower diagonal. Correlation of categorical fixed effects was assessed using the global model fixed effects correlation matrix, where no strong correlations were detected (all values < 0.36 and > −0.14). [file peerj-04-2622-s006.jpg]
